# Wine and Grape Tannin Interactions with Salivary Proteins and Their Impact on Astringency: A Review of Current Research

**DOI:** 10.3390/molecules16032348

**Published:** 2011-03-11

**Authors:** Jacqui M. McRae, James A. Kennedy

**Affiliations:** 1 The Australian Wine Research Institute, PO Box 197, Glen Osmond SA 5064, Australia; 2 Department of Viticulture and Enology, California State University, 2360 E. Barstow Ave MS VR89, Fresno, CA 93740, USA; E-Mail: jakennedy@csufresno.edu

**Keywords:** astringency, condensed tannin, salivary proteins, wine

## Abstract

Astringency is an important characteristic of red wine quality. The sensation is generally thought to be produced by the interaction of wine tannins with salivary proteins and the subsequent aggregation and precipitation of protein-tannin complexes. The importance of wine astringency for marketability has led to a wealth of research on the causes of astringency and how tannins impact the quality of the sensation, particularly with respect to tannin structure. Ultimately, the understanding of how tannin structure impacts astringency will allow the controlled manipulation of tannins via such methods as micro-oxygenation or fining to improve the quality of wines.

## 1. Introduction

Tannins, including grape-derived condensed tannins (flavonoids) produce sensations of astringency in food and drink and form the ‘structure’ or ‘body’ of red wine. The term astringency refers to the drying and a puckering sensation in the mouth [1] and is a characteristic of red wine and its mouth-feel [2,3,4,5]. Wine tannin quality is dependent on the maximum intensity of the mouth feel, total duration and time taken to reach maximum intensity [6], as well as the extent of mouth drying and mouth roughness [1,7,8]. The spectrum of subtle differences in astringency sensations was compiled as a ‘red wine mouth-feel wheel’ by Gawel *et al.* [9], which include such descriptors as ‘powder’ through to ‘adhesive’ and ‘aggressive’. Astringent sensations of wine are considered pleasant when balanced with other factors including alcohol and sugar content. Higher concentrations of tannins and acids compared with sugar results in a highly astringent wine that is considered ‘harsh’, ‘unripe’ or ‘green’, and conversely, higher concentrations of sugars can result in a wine that may be described as ‘thick’ or ‘flabby’ [10]. Astringency influences the quality of red wine [11,12] and therefore knowledge of the structures of astringent compounds in a wine matrix and the impact of these structures on the sensory properties can be an important aspect of winemaking. 

Tannins characteristically have a propensity to bind to proteins and therefore can potentially cause gastrointestinal problems by denaturing digestive enzymes in the gut. Salivary proteins are believed to bind efficiently to tannins to offset this effect and also to act as a detection mechanism [13,14,15]. The resulting aggregation of the protein-tannin complexes and subsequent increase in friction is generally thought to give rise to the sensation of astringency [14,16], however these interactions are only part of the complex sensation that can give a range of perceptions from a velvety smooth texture to a harsh, puckering sensation [9,17,18].

The astringency of wine is influenced by a number of factors, including the structures and quantity of the tannin in wine [19], the presence of macromolecules such as polysaccharides [20,21] and residual sugars [22], the concentration of smaller molecules such as anthocyanins and catechin monomers [7,23], the acidity [24,25], and ethanol concentration [8,24]. Ultimately the understanding of how different wine constituents contribute to astringency will enable growers and winemakers to have more control over the characteristics of the produced wine.

## 2. Mechanisms of Astringency

Astringency is a complex process involving many mechanisms and is generally considered to be a tactile sensation caused by a loss of lubricity in oral saliva [6,26]. The exact mechanisms of this process are not well understood, yet many factors are known to contribute to an astringent sensation, including an increase in friction [26,27], interactions between tannins and oral epithelial proteins [28] or with taste receptors, particularly bitter receptors in the case of small condensed tannins [6,7,29], and a change in saliva viscosity [30]. These factors are summarized here and are covered in more detail in the review by Bajec and Pickering [31].

The main mechanism behind the loss of saliva lubricity is thought to result from the interaction of astringent agents, such as tannins, with salivary proteins and glycosaminoglycans (mucopolysaccharides). The subsequent aggregation and precipitation of the protein-tannin complexes has been shown to reduce the lubricity of saliva by increasing friction in the oral cavity [14,16,32]. This process causes a drying and grainy sensation in the mouth and the sensation has been shown to differ based on the size and concentration, as well as the hardness or softness, of the precipitate [27,33]. The formation of soluble aggregates between hydrolysable tannins and gelatin *in vitro* has also shown to produce an astringent sensation *in vivo*, suggesting that precipitation is not a necessary to induce an astringent sensation [34]. Any remaining unbound tannins may interact with other oral surfaces, and experiments by Payne *et al.* [28] have demonstrated that tannins also interact with oral epithelial cells. This interaction was increased at lower pH, which correlated directly with an increase in perceived astringency. Further, tannins and their analogues may degrade in solution [35] and thus interact with taste receptors, particularly the bitter receptors [29,36]. 

The physiological response of the individual tasting the wine will also influence how they perceive the wine astringency. Salivary flow rate, viscosity and protein composition vary between people and the latter has been shown to have a significant effect on perceived astringency [8,14,37]. Higher concentrations of particular saliva proteins and a higher flow rate of saliva have been shown to generally reduce the sensation of astringency [6,37]. Saliva viscosity is not directly correlated with oral lubrication, however the sensation of astringency can be produced by a decrease in saliva viscosity, which thus increases friction [27]. 

Astringency involves many factors, however the key element in the development of this sensation is that astringent agents, particularly tannins, interact with proteins in saliva. The exact composition of saliva varies among individuals, it is generally reported as comprising mostly of proline-rich proteins (PRPs) as well as histidine-rich proteins (histatins or HRPs), α-amylase, lactoferrin, and mucin-glycoproteins [13,31,37,38], with PRPs and HRPs being the main tannin-binding proteins. Details of other tannin-binding salivary components are described in a review by Bennick [39]. PRPs are intrinsically unfolded proteins consisting of multiple tandem repeats, which provide numerous binding sites for interactions with tannins [40,41,42,43], particularly those amino acids that form part of a polyproline (PPII) helix [44]. 

PRPs are sub-classified as either basic, acidic or glycosylated proteins based on differences in amino acid sequences [21,31]. Basic PRPs amount to around 23% of PRPs in human parotid saliva and are involved directly with binding to food tannin to prevent it from inhibiting digestion enzymes in the stomach [39,43]. The amino acid sequences of many of these proteins have been determined and IB5 in particular has been widely used as a model PRP for many tannin-protein interaction studies due to its low complexity and representative structure [43,44,45]. Basic PRPs have been proposed as being the main tannin-binding PRPs [46], which may be due to the extended conformations of these proteins [47]. Acidic and glycosylated PRPs have also been found to bind to hydrolysable tannins [48], as well as flavanol polymers and monomers [49], suggesting that they may also contribute to the sensation of astringency. Acidic PRPs make up around 30% of known PRPs and are known to have a high affinity for binding with calcium and may therefore be part of the dental pellicle [39,46,48]. Glycosylated PRPs are the least abundant, incorporating only 17% of known PRPs and are responsible for oral lubrication and antibacterial activity [44]. Complexes of tannin and glycosylated PRPs have been shown to remain soluble, whereas complexes with non-glycosylated PRPs are more likely to precipitate from solution [50] although the impact of these findings on astringency is not known. 

.Histidine-rich proteins also important tannin-binding proteins in saliva, but only constitute 2.6% of salivary protein. They tend to be smaller than PRPs with histidine making up about a quarter of the amino acids present. Twelve different HRPs (HRP1-12) have been isolated from human saliva and their structures determined, with HRPs 1, 3 and 5 accounting for the vast majority of these proteins. All three HRPs have been found to bind and precipitate hydrolysable tannins, although HRP 1 demonstrated reduced capacity for precipitating condensed tannins compared with HRP 3 and 5 [51,52]. The extended structure and natively unfolded nature of PRPs and HRPs also allow access to binding sites more readily than proteins with more globular configurations including amylase [38,45]. The structures of salivary proteins, as well as those of the wine tannins, impact upon the extent of protein-tannin interactions in the oral cavity and thus influence the resulting astringency of wine.

## 3. Red Wine Tannins

Red wine tannins consist of condensed tannins extracted from grapes and subsequently structurally modified during wine-making. A small percentage of hydrolysable tannins are extracted from oak barrels or chips during aging [53], however these compounds alone are unlikely to contribute to astringency [54]. Condensed tannins from grape skins are extracted earlier in the fermentation process. As fermentation continues, tannins begin to be extracted from grape seeds and flesh [55,56]. Cold soaking of grapes has also been shown to increase the extraction of seed tannins in the absence of ethanol, which may be related to the softening of the seeds prior to fermentation [57]. 

**Figure 1:**
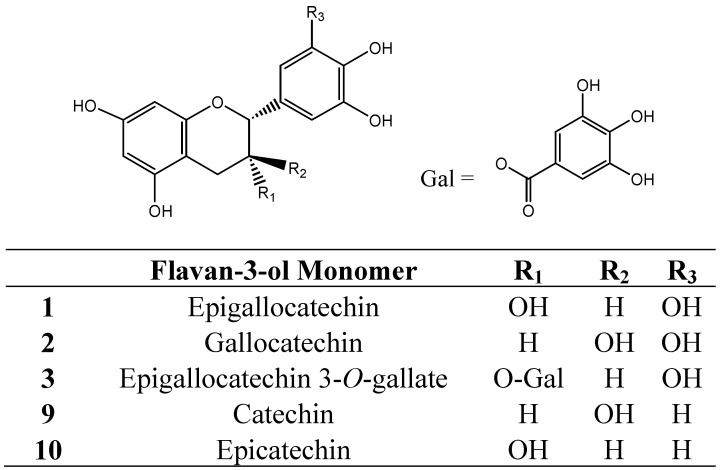
Structures of condensed tannin subunits (flavan-3-ol monomers)

**Figure 2:**
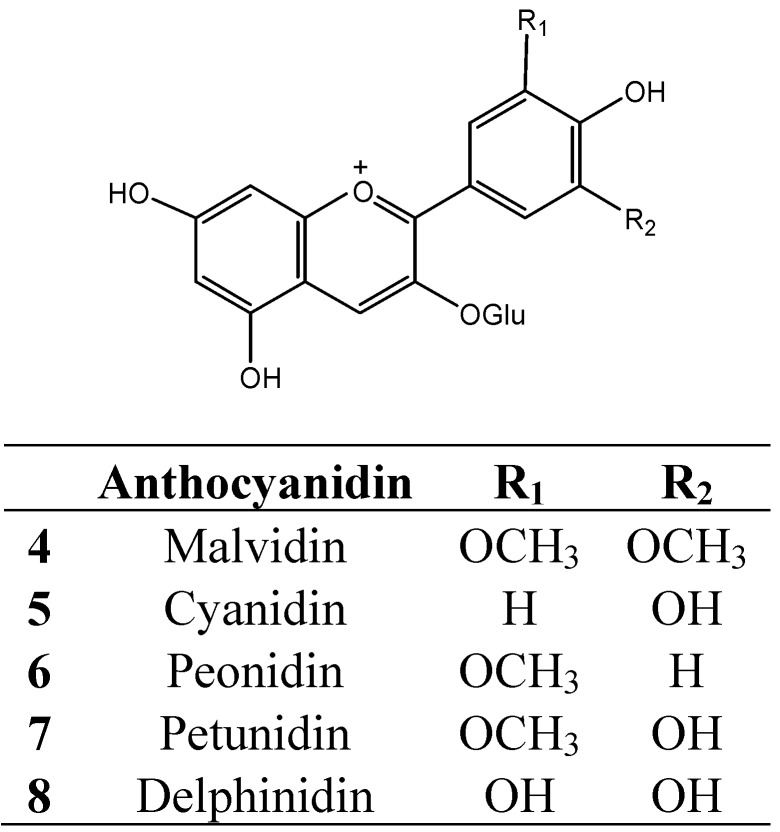
Structures of anthocyanins in wine.

Grape skin tannins consist of long polymeric chains ranging from 3 to 83 flavanol subunits (degrees of polymerization, DP) and are composed of procyanidins and prodelphinidins [29,58,59,60,61,62]. The tri-hydroxylated prodelphinidin subunits consist mainly of epigallocatechin (**1**, Figure 1), but with trace amounts of gallocatechin (**2**, Figure 1) and epigallocatechin 3-*O*-gallate (**3**, Figure 1) [62]. The tannin extracted from the skin of commercially ripe grapes consists of a portion of anthocyanins covalently bound to the oligomeric condensed tannins [63]. In the major winegrape varieties, anthocyanins include malvidin- (**4**), cyanidin- (**5**), peonidin- (**6**), petunidin- (**7**) and delphinidin- (**8**) 3-*O*-glucosides (Figure 2) [2,64], which may be incorporated into the structure of skin tannins. Seed tannins have a lower average degree of polymerization than skin tannins and are composed mainly of catechin (**9**, Figure 1) and epicatechin (**10**, Figure 1) subunits, with a greater proportion of galloylated units (13–29%) compared with skin tannins (3–6%) [65,66]. The size of seed tannins has been reported as between DP 2 to 17 [29,66,67,68]. The smaller molecular weight of seed tannins may be the reason for the reported bitterness of these compounds, and this may explain why seed tannins are considered undesirable in wine [65,69]. Flesh tannins exhibit greater molecular mass than seed tannins and comprise both epicatechin gallate and epigallocatechin subunits [68]. Grape stem tannins can contribute to the phenolic composition of wine and potentially increase the tannin concentration. The Dp of stem tannins ranges from 4 to 28, with a lower proportion of epigallocatechin subunits compared with epicatechin gallate subunits [70,71].

During fermentation, the structure of the extracted grape tannin is altered by enzymatic and chemical oxidation processes as well as indirect condensation reactions [64,65,72], which are facilitated by oxidation products such as acetaldehyde pyruvic acid and glycoxylic acid [73,74,75]. For example, acetaldehyde-mediated condensation reactions initially may involve the formation of ethyl-linked procyaninidin oligomers or pigmented polymers [76,77]. These can further polymerize to form coloured tannins that are potentially more prone to folding and intramolecular bonding than the more linear structures of grape tannins [78]. Wine tannin structure is less understood than grape tannin structure, which is largely because the structure is more resistant to traditional methods of tannin analysis such as acid-catalysed cleavage of the interflavan bonds and subsequent thiolysis or reaction with phloroglucinol [78,79].

After fermentation, wine constituents continue to undergo chemical changes which influence the structure of the tannin content. The acidic and slow oxidative conditions in wine lead to bond breaking and rearrangement reactions [33,80], which are thought to cause the polymerization of tannins, as well as the formation of different pigments and pigmented polymers [81,82,83,84]. Tannins from aged wines also have a greater quantity of coloured anthocyanins incorporated into the structure than tannins isolated from young wines [79] and this, to at least some extent, accounts for the decrease in anthocyanin concentration in wine with aging [85,86]. Oxidized tannins have been shown to feature greater intramolecular interactions, altering the conformation of the tannin in solution to more condensed or folded structures rather than the extended forms of grape tannins [78]. The changes in tannin structure with grape fermentation and wine aging are likely to impact upon the binding of the tannin with salivary proteins and thus the astringency of the wine.

## 4. Protein-Tannin Interactions and Astringency

From model studies it has been shown that tannins bind to proteins in three distinct stages. Initial interactions involve both hydrophobic interactions and hydrogen bonding and result in the formation of protein-tannin complexes. Hydrophobic interactions are entropy-driven and include Van der Waals interactions or π-π stacking of the electron-rich phenol ring of the tannin B-ring or galloyl ester and the planar *pro-S* face of the heterocyclic amide bonds in proline [16,41]. Hydrogen bonding is an enthalpy-driven electrostatic interaction that occurs between the tertiary amide or carbonyl groups of a proline subunit of a PRP [15,87] or the histidine imidazole ring or terminal carbon of an HRP, and the tannin hydroxyl groups [52,88]. The hydroxyl groups on the aromatic rings of condensed tannins have an acidic proton that acts as a proton donor, and a lone electron pair on the plane of the aromatic ring that functions as a proton acceptor. The ability of the tannin to bind to multiple sites on the randomly-coiled protein condenses the protein-tannin complex and making it more spherical [40].

The second stage of interaction involves the formation of protein aggregates with bound tannins, through self-association, causing cross-links between protein-tannin complexes [24,40,43]. The third stage of interaction occurs when the protein aggregates eventually coalesce producing colloidal particles that lead to precipitation of protein-tannin complexes, [14,40]. These processes have been shown to involve hydrogen bonding [72,87,89]. The concentration of salivary proteins affects the initial protein-tannin interaction, while environmental factors including pH, ionic strength and temperature influence the precipitation of formed aggregates in the second and third stages of interaction [90]. Differences in the size and hardness of the precipitate can impact upon the perceived astringency [22]. 

Greater concentrations of tannins have been shown to correlate directly with increases in perceived astringency [3,19], and differences in tannin structures have been shown to have a substantial impact upon the efficacy of protein binding as well as the perception of astringency [22,91,92]. Enhanced protein binding has been reported from tannins of greater molecular size and structural flexibility, containing a greater proportion of catechin subunits to epicatechin or epigallocatechin subunits, and more C4-C8 bonds than C4-C6 bonds [20,41,93,94]. Larger tannins with greater structural flexibility, such as freely rotating interflavan bonds and gallate groups, have a greater propensity to bind to proteins due to a larger number of available binding sites for interaction with the proline or histidine residues [16,65,95,96]. The increased size of the tannin also permits greater self-association, thereby promoting complex aggregation. The correlation between tannin size and efficacy in protein binding has been shown to have an upper limit depending on the tannin structure, since steric hindrance can prevent access to binding sites and greater molecular weight may limit solubility [16,97]. The presence of anthocyanins in the tannin structure also reduces the protein-binding capacity of the compound [65], although pigmented polymers have also been found to be positively associated with a puckering sensation [11,22]. 

**Figure 3:**
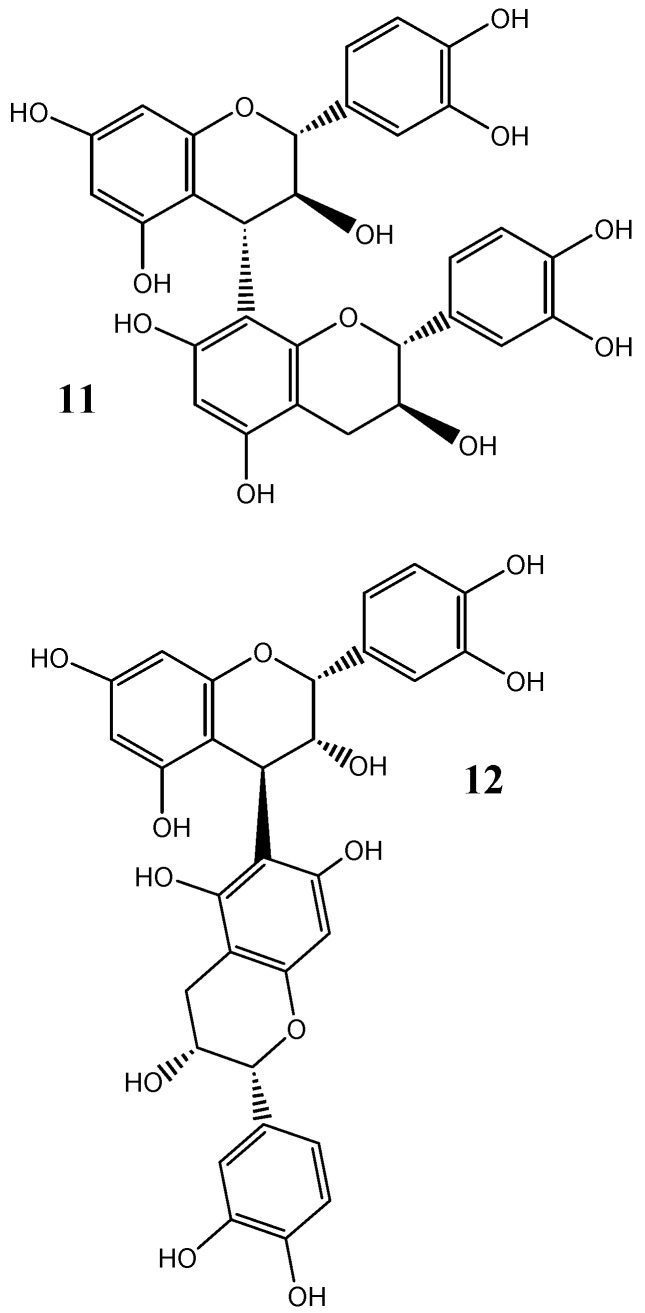
Structures of procyanidin dimers B3 (**11**) and B5 (**12**).

The conformation of tannins in solution has been shown to substantially affect the protein-binding efficacy of tannins. Flavan-3-ol subunits linked through a C4-C8 bond, such as procyanidin B3 (**11**, Figure 3). This results in a comparatively extended and more linear structure than C6-C8 bonds, such as procyanin B5 (**12**, Figure 3), which potentially enables greater interaction with more binding sites proteins rather than greater intra-molecular bonding [38]. Further, the stereochemistry of the 4-8 interflavan bond is dependent on the flavan-3-ol isomer of the upper subunit, relating to either a catechin derivative or epicatechin derivative, respectively. Catechin subunits reportedly have a higher specificity for PRPs relative to the epi-isomer, and these results also correlate with an increase in the perception of astringency for catechin compared with epicatechin [7,38,98], further demonstrating the stereo-specific binding nature of condensed tannins. The subsequent stereochemistry of the interflavan bond between these subunits may also influence the conformation of the formed polymer, resulting in the dominance of either an extended or compact form [99,100]. Both configurations have been shown to have a strong affinity for PRPs, with extended polymers promoting the formation of aggregates and compact polymers demonstrating greater hydrophilic interactions [72,87]. 

The aging of wine gradually alters the purple hue of young wine to brick-red and is considered to render the tannins less astringent. The change in hue is related to the formation of more stable pigments such as vitisin A and B and their derivatives from grape anthocyanins [101,102], as well as oxidative browning [103]. The cause for the decrease in astringency of red wine over time remains uncertain. A decrease in tannin concentration as a result of fining by residual proteins or polysaccharides [104], polymerization and subsequent precipitation, or conversely, depolymerization of tannins may contribute to the reduction in astringency [35]. However, some aged wines reportedly have similar concentration of tannin as young wines [3] and yet aged wines are generally considered to be less astringent, which suggests that tannin structural changes may also impact upon the perceived astringency [105]. Aged wine tannins have been shown to be larger than young wine tannins [79], a characteristic that is generally correlated with greater astringency [91]. Therefore it is possible that increased intramolecular bonding due to oxidation results in reduced structural flexibility and thus protein interaction, which may impact the astringency of aged wines. Artificially oxidized tannins have also been shown to have greater hydrophobicity than ‘native’ tannins [33], which may also impact upon the binding efficacy.

Micro-oxygenation (MOX) involves the controlled addition of small amounts of oxygen to a wine system either during fermentation or in the initial months post fermentation [106]. The effect of the increased oxygen exposure on red wine may contribute to a stabilization of wine colour and improved flavor and aroma [107,108,109]. One of the impacts of MOX treatment may be to produce changes in tannin structure that mimic the changes produced during aging, thus changing the perceived astringency of the wine [108,110]. The long term impacts of MOX on red wine are still being investigated.

## 5. The Impact of the Wine Matrix on Astringency

The interaction of wine tannin with salivary proteins, and the size and stability of the resulting protein-tannin complexes, are also dependent on other parameters of the wine matrix, particularly the pH and ethanol concentrations. Additional factors, including the concentration of organic acids, sugar, available acetaldehyde concentration, viscosity, and the presence of other compounds that interact with tannins such as residual yeast proteins and grape polysaccharides, can also impact upon the perception of astringency. The serving temperature of wine was found to have a minimal impact on the sensation of astringency [111]. 

Ethanol concentration varies in red wine from approximately 11% to 15% and higher concentrations have been shown to decrease the perception of astringency in model wines [24,58] and alter the astringency sub-qualities of wine [8], although one report indicated an increase in astringency with ethanol concentration [112]. A decrease in astringency with increasing ethanol concentration may at least in part be due to the conformational changes of tannins in higher ethanol wines. This may reduce the binding of tannins to proteins as well as the self-association of bound tannins, limiting the formation of protein aggregates [24]. Higher ethanol concentrations in model wine has also been shown to decrease the formation of protein-tannin aggregates [49,113]. Increasing ethanol concentration between 10 and 20% has also been shown to disrupt hydrophobic interactions between tannins and apple cell wall material, particularly for high molecular weight compounds with a higher degree of galloylation [98]. Further, greater ethanol concentrations may also increase the lubricity of the oral cavity, reducing the perception of roughness [8,24]. An increase in protein precipitation with ethanol concentration of 13% compared with aqueous solutions may relate to the change in solubility of the formed protein-tannin complexes [112]. Zanchi and colleagues demonstrated differences in ethanol solubility in a mixture of grape-seed tannins due to both self-aggregation [114,115] and PRP-tannin aggregation [33], which is likely to relate to differences in the structure of the tannins. Changes in solubility of tannins in wine may also influence the resulting astringency [115]. Finally, an increase in viscosity of the solution with greater ethanol content may also decrease the perception of astringency as well as protein-tannin interactions [8,30,89].

The pH of wine generally ranges from pH 3.2 to 3.8 and this difference is sufficient to elicit changes in astringency. Lowering the pH of wine and model wine solutions has been shown to increase the intensity of astringency as well as increase the association of tannins with proteins [24,116]. This effect is more significant than increasing the concentration of individual organic acids such as malic, lactic and tartaric acid [24,25], however greater organic acid concentrations combined with greater acidity have been shown to contribute to the chalky characteristics of red wine [22]. A combination of low pH and high organic acid concentration was also shown to be responsible for increasing the astringency of fermented coconut sap [117]. 

Tannins have been shown to bind to residual proteins or polysaccharides in the wine matrix, thereby reducing the concentration available for salivary protein interaction and thus reducing astringency [118]. This has been demonstrated in fruit, with the decrease in astringency of ripening fruit attributed to an increase in polysaccharides rather than a decrease in tannin concentration [32,119]. Different polysaccharides reduce the astringency of tannins by different mechanisms of action. Arabic gum and β-cyclodextrin preferentially bind to polyphenols, inhibiting protein-tannin interactions, while the polyelectrolytic properties of pectin enable it to bind directly to protein/polyphenol complexes, thereby increasing the water solubility of these complexes and preventing them from precipitating out of solution [21,120,121,122]. The polysaccharides in wine are classified based on their net charge, either neutral or acidic. Neutral polysaccharides in wine include arabinogalactan and pectin polysaccharides from grape cell walls and mannoprotein from the yeast during fermentation, and the main acidic polysaccharide is rhamnogalacturonan II. All polysaccharides have been shown to reduce the perception of astringency by some degree, however the acidic polysaccharides have shown a greater impact on astringency reduction [20,21,66,119]. The concentration of ethanol and ionic strength of the solution have also been shown to impact tannin-polysaccharide interactions as well as tannin-protein interactions [122].

Higher concentrations of sucrose and anthocyanins in wine have been associated with lower astringency ratings in wines and reducing the unpleasant ‘puckering’ sensation of young wines [22,123,124,125]. The use of the sweetening agent, aspartame, however, had no impact on the perceived astringency of model wine, suggesting that the reported association between high sucrose concentrations and reduced astringency may have been due to the increased viscosity of the solution [30]. The presence of oxidizing agents that promote polymerization such as acetaldehyde and glycoxylic acid have been shown to increase the perception of astringency, presumably due to the increase in tannin size [23]. Ethyl-linked flavan-3-ol dimers formed from reactions with acetaldehyde have been shown to have the same astringency as non ethyl-linked flavan-3-ol dimers [23]. The interactions of these factors in the wine matrix as well as differences in tannin structures and concentrations, all impact upon the perceived astringency of red wine. 

## 6. Conclusions

There has been considerable research into the mechanisms involved in wine astringency perception, the factors affecting astringency in wine and the structures of some of the contributing tannins and yet there is still a great deal to understand with respect to how the tannins present in grapes relate to wine tannins and astringency in the corresponding wine. Greater knowledge of the structure/ function relationships in protein binding, and knowledge of how tannin structure can be selectively changed to improve astringency would have important implications in winemaking.
